# Effects of terrorist attacks on access to maternal healthcare services: a national longitudinal study in Burkina Faso

**DOI:** 10.1136/bmjgh-2020-002879

**Published:** 2020-09-25

**Authors:** Thomas Druetz, Lalique Browne, Frank Bicaba, Matthew Ian Mitchell, Abel Bicaba

**Affiliations:** 1 Social and Preventive Medicine, University of Montreal, Montreal, Québec, Canada; 2 Centre de recherche en santé publique, Montreal, Québec, Canada; 3 Center for Applied Malaria Research and Evaluation, New Orleans, Louisiana, USA; 4 Société d'Études et de Recherches en Santé Publique, Ouagadougou, Burkina Faso; 5 Political Science, University of Saskatchewan, Saskatoon, Saskatchewan, Canada

**Keywords:** maternal health, health services research, other study design, public health

## Abstract

**Introduction:**

Most of the literature on terrorist attacks’ health impacts has focused on direct victims rather than on distal consequences in the overall population. There is limited knowledge on how terrorist attacks can be detrimental to access to healthcare services. The objective of this study is to assess the impact of terrorist attacks on the utilisation of maternal healthcare services by examining the case of Burkina Faso.

**Methods:**

This longitudinal quasi-experimental study uses multiple interrupted time series analysis. Utilisation of healthcare services data was extracted from the National Health Information System in Burkina Faso. Data span the period of January 2013–December 2018 and include all public primary healthcare centres and district hospitals. Terrorist attack data were extracted from the Armed Conflict Location and Event Data project. Negative binomial regression models were fitted with fixed effects to isolate the immediate and long-term effects of terrorist attacks on three outcomes (antenatal care visits, of facility deliveries and of cesarean sections).

**Results:**

During the next month of an attack, the incidence of assisted deliveries in healthcare facilities is significantly reduced by 3.8% (95% CI 1.3 to 6.3). Multiple attacks have immediate effects more pronounced than single attacks. Longitudinal analysis show that the incremental number of terrorist attacks is associated with a decrease of the three outcomes. For every additional attack in a commune, the incidence of cesarean sections is reduced by 7.7% (95% CI 4.7 to 10.7) while, for assisted deliveries, it is reduced by 2.5% (95% CI 1.9 to 3.1) and, for antenatal care visits, by 1.8% (95% CI 1.2 to 2.5).

**Conclusion:**

Terrorist attacks constitute a new barrier to access of maternal healthcare in Burkina Faso. The exponential increase in terrorist activities in West Africa is expected to have negative effects on maternal health in the entire region.

**Key questions**

**What is already known?**

- Improvements in access to healthcare are fragile in politically unstable countries such as Burkina Faso, and are likely to disappear if new barriers are introduced or former barriers are reinstituted.
- The primary factors that continue to limit women’s access to healthcare after user fee abolition are distance to health facilities, low quality of care and informal costs; however, the extent to which insecurity generated by terrorist attacks presents a new type or barrier to maternal healthcare access remains under-investigated.

**What are the new findings?**

- Our findings suggest that terrorist attacks have immediate repercussions on different indicators of maternal care, notably the number of antenatal care visits, assisted deliveries and cesarean sections.
- This study also reveals that repeated attacks aggravate insecurity and are further detrimental to maternal healthcare access in affected regions.

**What do the new findings imply?**

- Regional insecurity needs to be recognised and investigated by the global health research community as a barrier to maternal healthcare and universal health coverage.

## Background

In the past few years, several countries of the Sahelian region have been afflicted by a rise in insecurity related to terrorist attacks. The list of affected countries includes Nigeria, Mali, Sudan, Niger, Chad and most recently Burkina Faso.^1^ Initially spared such attacks, since 2015 Burkina Faso has been afflicted by an increased number of violent events related to terrorism. A recent report notes that Burkina Faso suffered more jihadist attacks than any other country in the Sahelian region in 2019.^2^ These attacks are mostly (but not exclusively) located in the Northern and Eastern border areas of the country. Ouagadougou, the capital, is located in the centre of the country and has also been hit by attacks, particularly on military bases and places sought after by tourists or foreign workers. As a landlocked country where poverty is endemic (it ranked 182 out of 189 countries on the 2019 Human Development Index (HDI),^3^ Burkina Faso is suffering the political, economic, social, health and humanitarian consequences of these intensifying attacks.^4^

The potential impact of terrorist attacks on population health goes beyond direct consequences (ie, people killed, injured, displaced or traumatised). Like armed conflicts, these attacks can damage public health infrastructure and services; undermine water, electricity and food supply; increase poverty; impede vaccination campaigns; and deteriorate sanitation and transportation.^5–10^ In addition, terrorist attacks aim to generate a feeling of insecurity in the general population, whose negative impact on numerous health indicators has been demonstrated in the context of armed conflicts.^11 12^ For example, studies reveal that the availability and access to maternal healthcare services, including family planning and contraception, are likely reduced under high-intensity conflict conditions, possibly contributing to increased maternal mortality.^13–17^

This is particularly troubling in Burkina Faso, where despite noticeable progress over the last two decades maternal health remains one of the biggest public health issues. In 2015, a nationally representative survey estimated the maternal mortality rate to be 330 deaths per 100 000 live births, compared with 12 per 100 000 on average in the richest countries.^18 19^ A major cause of this burden is the limited and unequal access to quality healthcare, especially in rural areas.^20^ In order to improve coverage, the Government of Burkina Faso has gradually mitigated the cost of healthcare services, first in 2007 by reducing fees associated to assisted deliveries by 80%, then in 2016 by abolishing all user fees for maternal healthcare services.^21^ Studies have demonstrated the positive impact of these initiatives on many indicators of maternal health, including the volume of antenatal care (ANC) visits and assisted deliveries, as well as on health inequalities.^22 23^

However, as suggested by a recent study, the improvements in access to healthcare are fragile in Burkina Faso, and are likely to disappear rapidly if a new barrier is introduced, or if a former barrier is reinstituted.^24^ Studies have shown that the primary factors that continue to limit women’s access to healthcare after user fee abolition are distance to the health facility, low quality of care and informal costs.^25–27^ However, it is plausible that the insecurity generated by terrorist attacks in Burkina Faso acts as a new type of barrier to maternal healthcare access. This could undermine the government’s longstanding efforts to improve maternal and neonatal health.

To our knowledge, the presence of such an ‘insecurity barrier’ to maternal healthcare access has never been examined in Burkina Faso. Studies conducted in other Sahelian countries afflicted by terrorist attacks are scarce and provide only limited evidence on the topic.^28–30^ In particular, no studies have used longitudinal evaluation designs to measure the immediate effects of attacks. Rather, they typically use data from cross-sectional surveys that are not time specific to such events and are therefore subject to historical bias in interpretation, due to time lag and a small number of time points.^31^

The objective of this study is to assess the effects of terrorist attacks on maternal healthcare access by using a more granular, precise spatiotemporal framework. A quasi-experimental study was therefore designed to (1) assess the immediate effects of terrorist attacks on access to maternal healthcare in Burkina Faso and (2) evaluate the longitudinal effects in communes affected by incremental levels of insecurity, defined here by the cumulative frequency of attacks. Three key outcomes are investigated, namely ANC visits, facility deliveries and cesarean sections.

## Methods

### Security context in Burkina Faso

Burkina Faso is a landlocked country of ~20 million inhabitants located in West Africa, and surrounded by Mali, Niger, Benin, Togo, Ghana and Côte d’Ivoire. Between 1987 and 2014, the Republic was governed by Blaise Compaoré, a former military man who seized power in a *coup d’état*. Throughout this period, Burkina Faso was considered to be a relatively secure country despite human rights violations and sporadic tensions and clashes between ethnic or religious groups. However, the security situation changed rapidly in the mid-2010s. After mounting pressure against his attempt to modify the Constitution in order to remain in power, Compaoré was forced to resign and flee the country.^32^ Presidential elections were organised in 2015, but not before the failure of a 1-week-long *contre-coup*. During this short period of unrest, approximately 15 people were killed and over 300 were wounded according to press releases.^33^

Meanwhile, the security situation had dramatically deteriorated in the neighbouring countries of Mali, Niger and (Northern) Nigeria, where jihadist groups—sometimes allied with rebel movements with territorial claims—carried out regular attacks against both the population and military forces.^34^ With these groups moving across borders and pursuing regional ambitions, the exact reasons that Burkina Faso remained relatively free of terrorist attacks remain unclear. Nevertheless, its government agreed in 2014 to enter the G5 Sahel Joint Force, along with Mauritania, Mali, Niger and Chad, to coordinate a regional response to the terrorist threat. Since then, several jihadist groups have escalated their attacks throughout the country, most notably Ansarul Islam, Islamic State in the Greater Sahara, and the Group to Support Islam and Muslims (known by its Arabic acronym JNIM). As a member of the G5 Sahel Joint Force, Burkina Faso’s military and police are supported in the field by Operation Barkhane, a French-led military force of approximately 5000 soldiers.

### Study design

This is a longitudinal quasi-experimental study that used multiple (pooled) interrupted time-series analysis to evaluate the effects of terrorist attacks on access to maternal healthcare services at the level of the lowest administrative unit (ie, the commune).^35^ Immediate effects were defined as level changes in the month of or the month following an attack. Longitudinal effects of repeated attacks were examined by defining segments based on the incremental number of attacks in a commune over time and by measuring level change between segments. All communes of the national database were included in the analysis. The study period spanned from January 2013 to December 2018, totalling 72 time points of observation.

### Outcome and exposure variables

This study has three outcome indicators: (1) the total number of ANC visits per commune per month; (2) the number of facility-based deliveries per commune per month; (3) the number of cesarean sections per commune per month. These outcomes were selected because they are key indicators of accessibility to maternal healthcare in low-income and middle-income countries and they are routinely collected in the facilities at the primary care level, including cesarean sections performed in district hospitals.^36^ In communes with several health facilities, the outcomes refer to the total number per commune per month. Models were adjusted for the proportion of missing data.^37 38^

Exposure was operationalised differently according to the objective. To evaluate the average immediate effects of a terrorist attack, communes that recorded at least one attack were defined as being exposed for that particular month and the following one, in order to cover a 30-day period after the attack. Therefore, the first exposure variable is categorical (no attack, single attack, multiple attacks) and reflects immediate exposure to an attack. Three categories were defined (rather than two, that is, absence/presence) to verify the presence of a dose–response relationship since it is hypothesised that more attacks will generate more insecurity and further reduce visits to health facilities. To evaluate the longitudinal effects of the incremental levels of insecurity, exposure was defined based on the cumulative number of attacks in a given commune over time. Exposure variable is therefore numeric and reflects the shift into a new ‘phase’ characterised by one additional attack. The duration of these phases (segments) vary since they last until a new attack occurs. For both objectives, a terrorist attack was defined as an act involving a jihadist group in which one of the protagonists used violence (ie, battle, explosion/remote violence, looting/property destruction and violence against civilians). Attacks involving ‘unidentified armed groups’ were included in terrorist attacks.

### Data sources

Two secondary sources of data were used. First, the utilisation of healthcare services data was extracted from the National Health Information System in Burkina Faso. Data were available from January 2013 to December 2018, which constitutes a reliable time series of 72 points of observation. All public facilities at the primary care level were considered in the analysis, that is, primary healthcare centres (‘Centres de santé et de promotion sociale’) and district hospitals (‘Centres médicaux avec antenne chirurgicale’). Every month, facilities review their record books and complete a form that is sent to the Health District, which compiles data from all the facilities in its catchment area. Data quality is assessed in each district before being transmitted to the Director of Health Statistics at the Ministry of Health, where data from all health districts are compiled. The Ministry of Health performs regular supervision visits and audits in the field. The data collection instruments (record books, monthly reports, national database structure) remained constant during 2013–2018. Data from the passive surveillance system in Burkina Faso have been proven reliable in previous studies.^39 40^

Second, terrorist attack data were extracted from the Armed Conflict Location and Event Data (ACLED) project. The ACLED project collects data on violent events within States, which includes armed conflicts and terrorist activities with or without fatalities. Data are disaggregated by date, location and actors. This spatial scale is relevant for the purpose of the present study since its hypothesis is that terrorist attacks reduce access to the surrounding primary care facilities, rather than at the national level.^41^ For those violent events with fatalities, ACLED data were cross-checked and completed by using the Uppsala Conflict Data Program Georeferenced Event Dataset (UCDP-GEP).^42^ Based on the GPS coordinates of the events, communes were identified by using the database of Global Administrative Areas (GADM). Finally, the ACLED and passive surveillance datasets were merged at the commune-month level of aggregation.

### Analysis

The unit of all analyses was the commune-month. To explore the attacks’ effects, three separate regression models (corresponding to the three outcomes) were fitted using the exact same set of variables and parameters. Even if the outcomes were all count variables, negative binomial regression was preferred over Poisson because of overdispersion. In order to best isolate the effect of attacks, the commune unit was entered as fixed effects while using unconditional maximum-likelihood estimation. This allows for control for any stable characteristic of the communes, whether observed or not.^43^ The underlying equation of the basic fixed effect level can be expressed as y_it_=μ_t_ + βx_it_ + α_i_ + ε_it_ with i=1, … n (communes) and t=1, … t (time) where μ is a constant term, y_it_ is the response value for the commune i at time t, x is a vector of time-variant variables, α_i_ are commune-specific intercepts that capture heterogeneity between communes and ε are residual errors.

Four time-varying variables were entered in the models: the monthly variation (calendar month), the baseline trend (time units since January 2013), the trend since occurrence of the first attack (time units since the month of the first attack in a commune) and the percentage of missing observations. The linearity of the relationship between the outcome and continuous covariate was assessed by adding quadratic terms. Multicollinearity was ruled out by using the Collin package (StataCorp, College Station, Texas) and verifying that variance inflation factors did not exceed 4. Robust variance estimators (Huber/White estimator) were used throughout the analyses. Coefficients were expressed as incidence rate ratios. The threshold for statistical significance was set at 0.05 (bilateral tests).

All analyses were performed in Stata V.14.0 (StataCorp). Maps were created using QGIS V.3.8 (open-source GIS software).

### Ethics

This study only uses secondary, administrative data. GADM, ACLED and UCDP-GEP data are publicly available online (https://gadm.org/, https://acleddata.com/ and https://ucdp.uu.se/). Access to the National Health Information System data was granted by the Ministry of Health of Burkina Faso (Notice #2018-3032).

#### Patient and public involvement statement

Patients and members of the public were not used in the design, conduct, reporting and dissemination of this research. Utilisation of healthcare services by patients were routinely collected by health facilities providers and analysed; however, data were aggregated and individual patients cannot be identified from the reported data.

## Results

The spatiotemporal structure of the national health information system database is described in table 1. It totalises 25 572 commune-months (the level of analysis), representing data from 356 communes (this includes some communal sub-sections of the two largest cities, Ouagadougou and Bobo-Dioulasso) in Burkina Faso over a 6-year period. Nearly all communes in Burkina Faso had at least one primary care facility in 2018, which makes the database nationally representative.^44^ During that period, there were a total of 388 violent events, of which 313 (81%) involved a jihadist group (see table 2).

**Table 1:** Description of the longitudinal database, by region

Region	Population (2015)*	Number of primary healthcare centres (2018)	Number of district hospitals (2018)	Number of communes†	Number of commune-months	Number of health districts
Boucle du Mouhoun	1 821 059	228	5	46	3312	6
Cascades	739 497	94	1	17	1224	3
Centre	2 532 311	123	5	11	792	5
Centre Est	1 470 903	144	6	30	2100‡	7
Centre Nord	1 547 565	149	3	28	2016	6
Centre Ouest	1 510 975	223	3	38	2736	7
Centre Sud	804 709	113	4	19	1368	4
Est	1 615 740	158	4	27	1944	6
Hauts Bassins	1 961 204	187	5	35	2520	8
Nord	1 502 527	216	4	31	2232	6
Plateau Central	875 910	141	3	20	1440	3
Sahel	1 272 545	106	3	26	1872	4
Sud Ouest	795 549	109	3	28	2016	5
Total	18 450 494	1991	49	356	25 572	70

*Ministère de la Santé. Annuaire statistique 2015. Ouagadougou: Ministère de la Santé du Burkina Faso, Direction générale des études et des statistique sectorielles; 2016 (http://cns.bf/IMG/pdf/annuaire_ms_2015_signe.pdf). Health facilities data were obtained from the Ministry of Health’s Système national d’information sanitaire. Administrative units information was obtained from Global Administrative Areas database. See methodology section for further description of data sources.

†Including some communal sub-sections (‘Arrondissements’) of Ouagadougou and Bobo-Dioulasso.

‡60 units are missing because the only health facility in Déguéba commune opened in January 2018.

**Table 2:** Violent events involving at least one non-State actor, by type and year, 2013–2018

	2013	2014	2015	2016	2017	2018
No of events	5	7	9	23	99	245
No of deaths	6	8	9	74	109	302
No of communes affected by ≥1 event	3	8	7	15	33	73
No of events involving a terrorist group and civilians	0	0	1	8	65	88
No of events involving a terrorist group and the armed forces	0	0	4	8	21	118
No of events involving the armed forces and civilians	1	1	2	1	6	26
No of events involving at least 1 militia or ethnic group	1	3	1	3	6	12
No of other events*	3	3	1	3	1	1

*Other events include clashes between civilians or between different factions of the armed forces.

**Figure 1:**
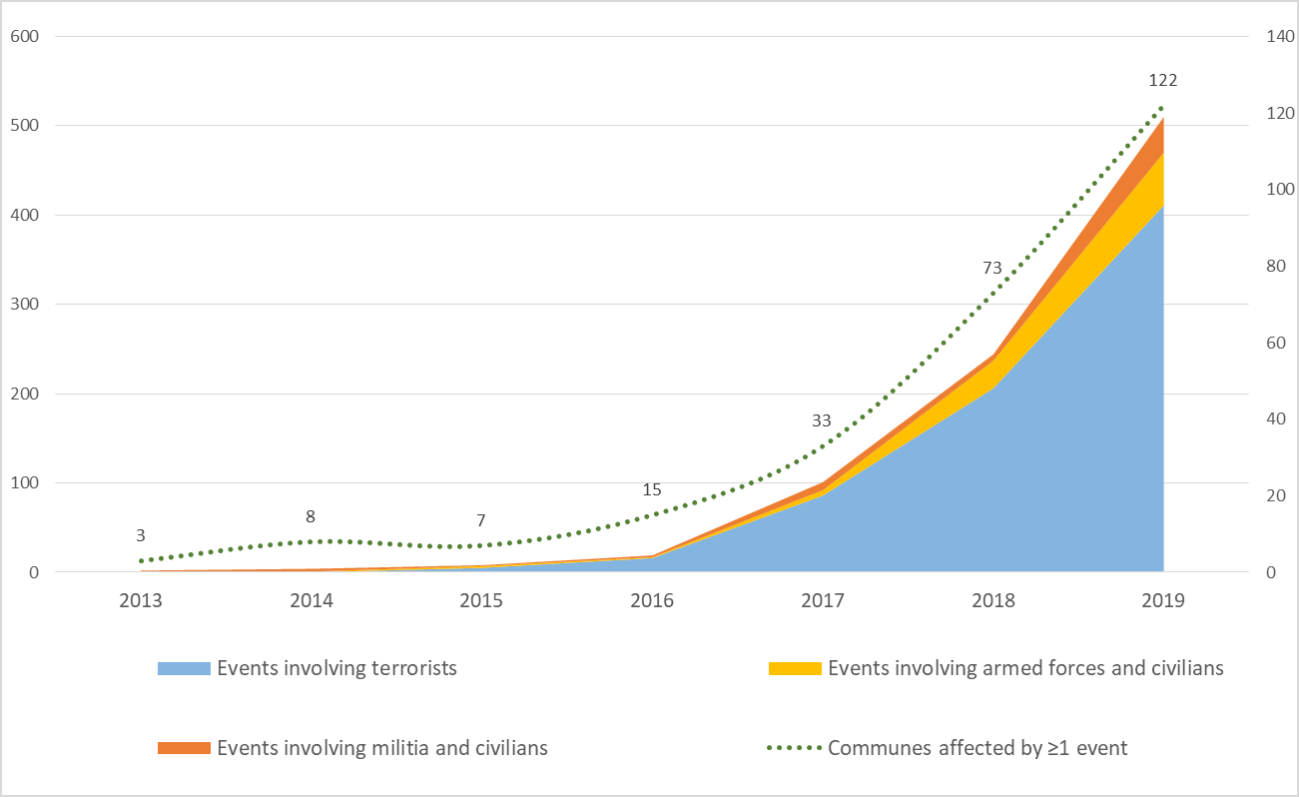
Trends in violent events in Burkina Faso, 2013–2019.

**Figure 2:**
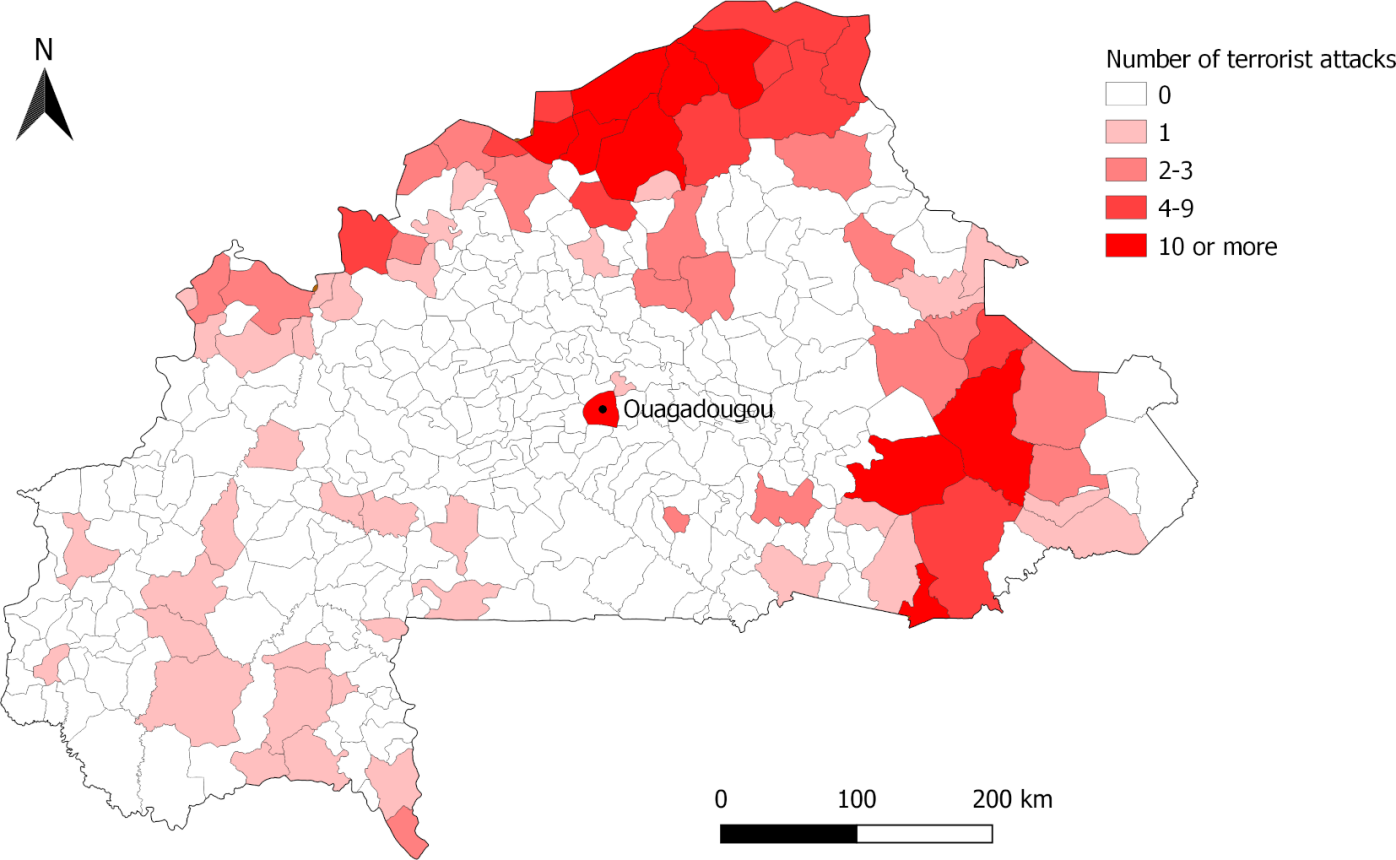
Map of the communes in Burkina Faso, by terrorist activity level, 2013–2018.

Terrorist attacks started in 2015 and have grown exponentially, reaching a total of 206 and 411 in 2018 and 2019, respectively (see figure 1). Other types of violent events (involving ethnic and self-defence militias, civilians and armed forces) have also been rising over the same period. The degree of violence of terrorist attacks (ie, the mean number of deceased individuals per attack) remained quite stable, ranging from 1.4 in 2015 to 0.85 in 2018. The two deadliest attacks occurred in Ouagadougou in January 2016 and August 2017, with 30 and 19 civilian deaths, respectively. The number of communes affected by terrorist attacks increased from 4 in 2015 to 73 in 2018, which represents ~20% of the total number of communes in the country (see figure 2).

The immediate effects of terrorist attacks on the three study outcomes are presented in table 3. Two different gradients in the effects estimates were observed. First, the effects of multiple attacks (per commune per month) were more severe than the effects of a single attack, regardless of the outcome. Second, the effects’ magnitude of attacks (single or multiple) was more important for cesarean sections than for assisted deliveries, which in turn was more important than for ANC visits. For example, the incidence of cesarean sections following a single attack was immediately reduced by 5.3% (incidence rate ratio (IRR) 0.947, 95% CI 0.819 to 1.088), while multiple attacks reduced it by 12.4% (IRR 0.876, 95% CI 0.723 to 1.060). For the assisted deliveries, IRRs were 0.962 (95% CI 0.937 to 0.987) and 0.904 (95% CI 0.861 to 0.950), respectively, while they were 0.998 (95% CI 0.972 to 1.024) and 0.927 (95% CI 0.881 to 0.974) for the ANC visits. Since the number of district hospitals that perform cesarean sections is more limited than the number of primary healthcare centres, it is not surprising that statistical tests reached significance only for the latter, even if effects are more pronounced for the former. For each of the three outcomes, the monthly trend in the phase following the first attack was statistically significantly <1. This suggests that trends in the number of cesarean sections, assisted deliveries and ANC visits are negatively affected once a commune experiences a terrorist attack.

**Table 3:** Effects of terrorist attacks in Burkina Faso on three reproductive health outcomes*

	Cesareans		Assisted deliveries		Antenatal care visits	
	IRR	95% CI	IRR	95% CI	IRR	95% CI
Terrorist attack during the month						
Zero (ref)	1		1		1	
Single	0.947	0.819 to 1.088	0.962**	0.937 to 0.987	0.998	0.972 to 1.024
Multiple	0.876	0.723 to 1.060	0.904***	0.861 to 0.950	0.927**	0.881 to 0.974
Baseline trend	1.009*	1.002 to 1.017	1.001***	1.001 to 1.002	1.004***	1.003 to 1.004
Quadratic term for baseline trend	0.999	0.999 to 1.000	0.999***	0.999 to 0.999	0.999***	0.999 to 0.999
Trend in attack phase	0.982***	0.975 to 0.989	0.996***	0.995 to 0.997	0.996***	0.995 to 0.997

*p<0.05; **p<0.01; ***p<0.001.

†Results are derived from three separate models (one per outcome) that were fitted using negative binomial regression with robust variance estimators and fixed effects at the commune level. The exposure variable is categorical and expressed by the number of attacks per month per commune (with three categories). The same set of covariates was used in each model, two of which (the month and the percentage of missing observations) are not displayed here. The number of observations for cesareans is smaller because they are only performed in reference health facilities.

IRR, incidence rate ratio.

**Figure 3:**
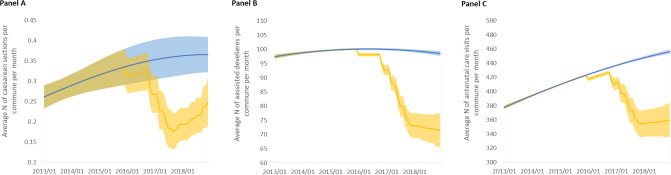
Predicted trends (with 95% CI) of the three study outcomes in the commune of Tongomayel, 2013–2018. Trends in blue are predictions with no attack in the commune (natural trends). Trends in orange are predictions based on the actual number and timing of attacks experienced by the commune of Tongoyamel. (A) Trends in the number of cesarean sections. (B) Trends in the number of assisted deliveries. (C) Trends in the number of antenatal care visits.

The segmented regression analyses further investigate the longitudinal effects of repeated attacks. They reveal that the incremental number of terrorist attacks, a longitudinal indicator of the cumulative insecurity in a commune, is negatively associated with the three outcomes (see table 4). For every additional attack in a commune, the incidence of cesarean sections is reduced by 7.7% in the next segment (95% CI 4.7 to 10.7). For assisted deliveries, incidence is reduced by 2.5% (95% CI 1.9 to 3.1), and for ANC visits, by 1.8% (95% CI 1.2 to 2.5). However, as suggested by the modest but statistically significant quadratic terms, the reduction is not constant and tends to lessen as the number of attacks increases. Models can therefore predict trends for each of the outcomes based on the number of terrorist attacks in a commune and their timing. Figure 3 displays the predicted trends of a commune that recorded 31 terrorist attacks over 34 months (which was the observed situation in Tongomayel, a commune located in northern Burkina Faso), as well as the natural trend that would have been observed in the absence of terrorist attacks.

**Table 4:** Longitudinal effects of repeated terrorist attacks in Burkina Faso on three reproductive health outcomes*

	Cesareans		Assisted deliveries		Antenatal care visits	
	IRR	95% CI	IRR	95% CI	IRR	95% CI
Cumulative frequency of terrorist attacks	0.923***	0.893 to 0.953	0.975***	0.969 to 0.981	0.982***	0.975 to 0.988
Cumulative frequency of terrorist attacks (quadratic term)	1.002***	1.001 to 1.003	1.001***	1.000 to 1.001	1.000**	1.000 to 1.000
Baseline trend	1.009^*^	1.002 to 1.017	1.001***	1.001 to 1.002	1.004***	1.003 to 1.004
Quadratic term for baseline trend	0.999	0.999 to 1.000	0.999***	0.999 to 0.999	0.999***	0.999 to 0.999

*p<0.05; **p<0.01; ***p<0.001.

†Results are derived from three separate models (one per outcome) that were fitted using negative binomial regression with robust variance estimators and fixed effects at the commune level. The exposure variable is numeric and expressed by the cumulative frequency of terrorist attacks per commune. The same set of covariates was used in each model, two of which (the month and the percentage of missing observations) are not displayed here. The number of observations for cesareans is smaller because they are only performed in reference health facilities.

IRR, incidence rate ratio.

## Discussion

Burkina Faso has implemented several measures over the past 10 years to increase its population’s access to healthcare services.^21^ It has been one of the first countries in sub-Saharan Africa to remove healthcare user fees for children under 5 years of age and for pregnant women.^45^ However, rising insecurity since 2015, mainly caused by terrorist attacks, is a major challenge to the achievement of universal health coverage. These findings have wider repercussions for other countries in the Sahel, given their endemic levels of poverty (6 of the 7 lowest ranked countries in the 2019 HDI are in the Sahel^3^) and their political instability and vulnerability to terrorist attacks.^46^ Moreover, the risk of jihadist contagion in West Africa could also undermine efforts in that region to improve healthcare services.^47^

To the best of our knowledge, this study is the first to document the presence (and assess the effects) of an ‘insecurity barrier’ to healthcare access. It shows that terrorist attacks have immediate repercussions on different indicators of maternal care, notably the number of ANC visits, assisted deliveries and cesarean sections. It also reveals that repeated attacks aggravate this insecurity and are further detrimental to healthcare access. All associations were in the anticipated direction and two gradients in the effects were observed.

The first gradient that was observed concerns the effects’ magnitude and bears serious clinical significance. Indeed, the reduction in healthcare services was moderate for ANC visits, which is likely due to the fact that these visits happen during daytime hours and can easily be rescheduled. On the other hand, the reduction was more pronounced for assisted deliveries and for cesarean sections, which are critical care seeking and treatment practices to reduce maternal and neonatal mortality. This larger effect for the most proximal indicators of maternal health could partly be explained by the fact that deliveries and obstetric emergencies can take place at night, when insecurity is maximal. Women may decide to remain and deliver in their village, especially if the nearest primary care facility no longer operates during night-time hours. Other explanations might include disruptions of the healthcare system engendered by the attacks, such as material stock-outs, staff absenteeism or lack of medical transportation to district hospitals. Indeed, terrorist attacks can affect both demand for and provision of healthcare services. Insecurity may likely encourage health staff to leave the affected areas or to reduce their activities, as suggested by reports of an increasing number of non-functioning health facilities in the country.

The second gradient that was observed is similar to a dose–response relationship. Regardless of the outcome, the immediate effects of a single attack in a particular month were smaller than the immediate effects of multiple attacks. Indicative of a severely insecure environment, the occurrence of multiple attacks in a single commune in 1 month was significantly associated with a reduction in the number of ANC visits and of assisted deliveries. The reduction in cesarean sections was even larger, but not statistically significant due to the small number of communes that have district hospitals and were subject to terrorist attacks. The number of attacks per month was preferred over the number of victims per month as an indicator of more intense exposure because data about the latter are harder to validate and likely in collinearity with population density.

Finally, the longitudinal analysis shows that the insecurity level in a commune is negatively associated with the use of maternal healthcare services. Successive terrorist attacks have an incremental effect; for every additional attack, a new segment can be defined where the average number of ANC visits, assisted deliveries and cesarean sections is significantly lowered. This reduction is not constant; rather, as the number of attacks increases, the effects tend to be of reduced magnitude, but levels remain significantly lower than those of the counterfactual (the hypothetical situation that communes would have known in the absence of terrorist attacks).^48^

### Strengths and limitations

This study is a natural experiment that relies on secondary data and, as such, is subject to some limitations. Data about the occurrence of attacks in remote areas can be difficult to validate and of inconsistent quality. In particular, errors of misclassification are likely. For example, violent events that concerned armed forces and civilians were excluded from the analysis, while it is possible that the armed forces engaged with individuals who were presumed (but not confirmed) terrorists. Also, spatiotemporal information about the events may be inaccurate.^49^ Several measures were taken to reduce such information bias. First, where possible, terrorist attacks data were corroborated between two databases. Second, data were aggregated at the higher spatiotemporal levels (ie, the commune-month) and the analysis was ecological.

Data from the health surveillance system are subject to non-random missingness since facilities located in areas with higher insecurity could be more prone to cease data entry in record books or cease transmission of information to the Health District. Even if analyses were adjusted for missing data at the commune level, some information bias is still plausible. Bilateral tests suggest no difference in missingness according to the occurrence of a terrorist attack or not.

Another limitation is the absence of measures at the individual level. In particular, the exposure variable in the longitudinal analysis relates to levels of insecurity that were defined independently from their perception by the community members. However, this was also the case for the outcome variables, all measured at the health facility level. Therefore, as stated previously, it is important to acknowledge that this is an ecological study that precludes drawing conclusions at the individual level. The impact of terrorist attacks and growing insecurity levels on the behaviour of pregnant women remains to be investigated.

Also, it was not possible to adjust the estimates for variations in populations at the commune level. Terrorist attacks likely urged some households to leave the affected areas—the number of internally displaced persons has been growing exponentially in Burkina Faso over the last few years. Arguably, these population changes could partly explain the negative long-term trends that were observed for each of the three outcomes in post-attack phases.

Several measures were taken to increase the internal validity of the effects evaluation in this quasi-experimental study. First, a robust design was used (pre–post with control group) and the conclusions were fuelled by three outcome indicators, following recommendations to use theoretical replication in evaluation studies.^31 50 51^ Second, analyses used multiple segmented regression with fixed effects that controls for time-invariant observables and unobservables at the commune level.^43 52^ With robust variance estimators, this longitudinal analysis is an evaluation design particularly appropriate to adjust for serial autocorrelation and selection bias.^53 54^ Finally, this study used a national dataset with a long observation period. These characteristics allow for a robust estimation of secular trends and considerably reduce the risk of historical bias, while the consistency of data collection instruments and aggregation methods throughout the observation period decreases the risk of instrumental bias.^35 55^

## Conclusion

Terrorist attacks constitute a new barrier to access of maternal healthcare services in Burkina Faso. They contribute to changes in delivery practices by reducing the number of ANC visits, assisted deliveries and cesarean sections in primary healthcare centres and district hospitals. The exponential increase in the number of terrorist activities in West Africa is therefore expected to have deleterious effects on maternal health in multiple countries and through different mechanisms. This problem could be compounded by the COVID-19 pandemic, which threatens to further strain the region’s already weakened health infrastructure, to increase inequalities and to reduce coordinated counterterrorist efforts. This, ultimately, could contribute to an upsurge of terrorist activity and increased insecurity across the Sahel.^56^ Perhaps more than ever, regional insecurity needs to be recognised and investigated by the global health research community as a barrier to universal health coverage. As for the wider crisis in the Sahel, the international community must remain steadfast in working to resolve the multidimensional problems that threaten the region.^57 58^
